# Comparative Genome Analysis of *Bacillus sporothermodurans* with Its Closest Phylogenetic Neighbor, *Bacillus oleronius*, and *Bacillus cereus* and *Bacillus subtilis* Groups

**DOI:** 10.3390/microorganisms8081185

**Published:** 2020-08-04

**Authors:** Rodney Owusu-Darko, Mushal Allam, Arshad Ismail, Carlos A. S. Ferreira, Sílvia D. de Oliveira, Elna M. Buys

**Affiliations:** 1 Department of Consumer and Food Sciences, University of Pretoria, Private Bag X20, Hatfield, Pretoria 0028, South Africa; rodneyowusu-darko@tuks.co.za; 2 National Institute for Communicable Diseases, Private Bag X4, Sandringham, Johannesburg 2131, South Africa; mushala@nicd.ac.za (M.A.); arshadi@nicd.ac.za (A.I.); 3 Laboratory of Immunology and Microbiology, School of Health and Life Sciences, Pontifícia Universidade Católica do Rio Grande do Sul (PUCRS), Porto Alegre 90619-900, Brazil; cferreira@pucrs.br

**Keywords:** heat shock, comparative genomics, protein cluster, *Bacillus sporothermodurans*, *Bacillus oleronius*, heat resistance

## Abstract

*Bacillus sporothermodurans* currently possesses one of the most highly heat-resistant spores (HRS), which can withstand ultra-high temperature (UHT) processing. Determination of multiple whole genome sequences of *B*. *sporothermodurans* provided an opportunity to perform the first comparative genome analysis between strains and with *B*. *oleronius*, *B*. *cereus*, and *B*. *subtilis* groups. In this study, five whole genome sequences of *B. sporothermodurans* strains, including those belonging to the HRS clone (SAD and BR12) normally isolated from UHT milk, were compared with the aforementioned *Bacillus* species for gene clusters responsible for heat resistance. In the phylogenomic analysis, *B. sporothermodurans*, with its closest phylogenetic neighbor, *B*. *oleronius*, clustered with *B*. *thermoamylovorans* and *B*. *thermotolerans.* Heat shock proteins GrpE, GroES, GroEL, and DnaK presented identical sequences for all *B*. *sporothermodurans* strains, indicating that differences in functional efficiency are not involved in the thermal resistance variations. However, comparing all species evaluated, *B*. *sporothermodurans* exhibited a different gene configuration in the chromosomal region of the heat shock protein GrpE. Furthermore, only *B*. *sporothermodurans* strains presented the stage II sporulation protein P gene located in this region. Multisequence alignment and phylogenetic analysis of the ClpB protein showed differences for HRS and non-HRS strains. The study identified ClpC, ClpE, and ClpX as the three ATPases putatively involved in protein disaggregation in *B*. *sporothermodurans*. *Bacillus* *sporothermodurans* exhibits high homology with other *Bacillus* species in the DnaK, DnaJ, GroEL, and GroES cluster of genes involved in heat resistance. The data presented here pave the way to select and evaluate the phenotypic effects of genes putatively involved in heat resistance.

## 1. Introduction

Comparative genome analysis typically consists of the identification of homologs (orthologues and paralogues), thus elucidating bacterial rearrangements, plasticity, epigenetics, and polymorphisms [1,2]. The afore-mentioned bacterial analysis has especially taken off since the gains in next generation sequencing (NGS) technologies [3,4,5]. Recently, NGS technologies evaluating whole genomes have made it possible to study the architecture of bacterial species, with the aim of identifying phylogeny and evolutionary trends [6,7,8].

Whole genome sequencing (WGS) of particularly industrially important organisms and bacterial pathogens provides critical answers for the biotechnology industry, human health, and wellbeing, as well as the environment. With the advent of bacterial bioprospecting, comparative genomics is the key to identifying new sources of important bacterial metabolites of benefit to the food, agriculture, health, and chemical industries. The study of evolutionary trends and pathogenesis of important disease-causing bacterial species would allow the development of new antibiotics and detailed knowledge of genetic mechanisms, along with the possible use of recombinant technology for pathogen control. Through NGS technologies, significant data are being generated, enabling the understanding of the moderate to high conservation of bacterial species via numerous proteins and unravelling the mechanisms of their adaptation to new niches and competition with other bacteria or forms of life [9].

The genus *Bacillus* comprises low G + C Gram-positive bacteria that are readily found in soil and water and, as such, can easily contaminate the food supply chain. Their ability to form spores in unfavorable conditions enables their survival in harsh environmental conditions that will, otherwise, kill vegetative bacteria. These unfavorable conditions include, but are not restricted to, wet and dry heat, extreme desiccation, ultraviolet and gamma irradiation, and oxidizing agents [10]. *Bacillus* spp. are of importance for the production of various exogenous enzymes such as amylase, protease, lipase, and phytase, used in detergents, starch hydrolysis, textiles, and animal feed applications [11]. *Bacillus* spp. have high growth rates and short cycles in industrial fermentations, making them good candidates for extracellular enzyme production [9]. Several species in this genus can ferment in acid, neutral, and alkaline pH ranges and are engineered to produce nucleotides, riboflavin, and ribose, amongst a variety of other industrial products. Industrially important *Bacilli* include *B. subtilis*, *B. licheniformis*, *B. amyloliquefaciens*, and *B. megaterium*. On the other hand, a few species, such as *B. cereus* and *B. anthrax*, infect humans, causing food-borne illness and anthrax, respectively.

*Bacillus sporothermodurans* is an emerging highly heat-resistant spore (HRS)-forming bacteria of interest to the dairy industry. HRS can survive in foods treated with ultra-high temperature (UHT), affecting quality and causing significant economic losses. After contamination of UHT milk products, these spores can germinate and grow in the stored milk, possibly reaching 10^5^ colony-forming unit (CFU)/mL. Taking into account that UHT milk products are considered commercially sterile when there is a total count of ≤10 CFU/0.1 mL, contamination with *B*. *sporothermodurans* may result in exceeding this limit. Even though *B*. *sporothermodurans* neither affects the pH nor the sensory quality and rarely causes any characteristic spoilage, its presence contravenes good manufacturing practice [12], because of the non-sterility of UHT milk products. Although no known pathogenic properties have been characterized to date, it is important to prevent its occurrence in UHT milk for quality purposes. Additionally, molecular approaches are needed to properly identify *B*. *sporothermodurans* and to differentiate strains isolated from UHT milk with likely higher heat resistance and those isolated from other sources including raw milk, milking equipment and feed concentrate [13]. Differences in heat resistance are thought to be clonal, which explains the growing interest in the identification of evolutionary associations and the understanding of its high heat resistance. In this study, the whole genomes of *B. sporothermodurans* strains isolated from UHT milk from South Africa and Brazil, the type strain, and *B. oleronius*, its closest phylogenetic neighbor, were sequenced [14,15], with the aim of comparing proteins involved in heat resistance between *B*. *sporothermodurans* and other *Bacillus* species to better understand this phenotype in *B*. *sporothermodurans*. By using molecular comparisons, we report various protein family clusters shared with other *Bacillus* species. To the best of our knowledge, this is the first study comparing *B. sporothermodurans* genomes with those of other *Bacillus* species.

## 2. Materials and Methods

### 2.1. Bacterial Isolation and Identification

*Bacillus sporothermodurans* strains SAD and SA01 were isolated from UHT milk sourced from local producers in South Africa, and *B. sporothermodurans* strain BR12 was isolated from UHT milk produced in Brazil. The type strains *B. sporothermodurans* DSM 10599 and *B. oleronius* DSM 9356 were obtained from the Leibniz Institute DSMZ—German Collection of Microorganisms and Cell Cultures GmbH (DSMZ). Growth of *B. sporothermodurans* isolates was performed on brain heart infusion agar (Hampshire, Oxoid, UK) and incubated at 37 °C for 48 h. *Bacillus oleronius* was grown on nutrient agar (Oxoid, UK) at 37 °C for 24 h. Genomic DNA was extracted using the ZR Bacterial DNA Miniprep kit (Zymo Research, Irvine, CA, USA). The DNA was quantified using the Qubit instrument and the dsDNA BR Assay kit (Life Technologies, Grand Island, NY, USA). Molecular confirmation of *B. sporothermodurans* strains and SA01 and BR12 as HRS was performed by PCR, as previously described [13,14].

### 2.2. Genome Sequencing and Analysis

*Bacillus sporothermodurans* strains SAD, SA01, BR12, and DSM 10599, as well as *B. oleronius* DSM 9356, were sequenced and annotated as previously described [15,16]. Subsequent analyses in this study are based on the strains mentioned above. *Bacillus sporothermodurans* B4102 and the selected *B. subtilis* and *B. cereus* group members were sourced from NCBI. Subsystem-based annotation was undertaken for all 12 *Bacillus* species, employing Rapid Annotation using Subsystem Technology (RAST) (Chicago, IL, USA) [17,18,19].

Genome sequences for *B. sporothermodurans* strains SAD, SA01, BR12, DSM 10599, and B4102 (accession numbers NAZD01000000, NAZB01000000, NAZA01000000, NAZC01000000, and LQYN00000000, respectively) and *B. oleronius* DSM 9356 (MTLA01000000) were used in this study. The *B. subtilis* group members included were *B*. *subtilis* subsp. *subtilis* str. 168 (CP019663), *B. licheniformis* DSM 13 = ATCC 14580 (CP000002), and *B. amyloliquefaciens* DSM 7 (FN597644). The *B. cereus* group members included *B. cereus* ATCC 10987 (CP026375), *B. anthracis* str. ‘Ames Ancestor’ (AE017336), and *B. thuringiensis* strain ATCC 10792 (CP020754).

Bioinformatics tools, including RAST and the Pathosystems Resource Integration Centre platform (PATRIC) (Chicago, IL, USA), were used to identify genes and protein families [17,20] of *B. sporothermodurans* strains, *B. oleronius* DSM 9356, and selected *B. subtilis* and *B. cereus* group members. Molecular Evolutionary Genetics Analysis (MEGA) v. 7.0 (University Park, PA, USA) was used for phylogenetic analysis [20,21,22].

A phylogenetic tree was constructed by comparing the conserved protein sequences obtained in this study with sequences of related *Bacillus* spp. selected from NCBI, using the programs RAxML (Randomized Axelerated Maximum Likelihood) (Heidelberg, Germany) [23] and FigTree v. 1.4.3 (Edinburgh, UK) [24]. The Phyre2 suite of tools was used to predict the tertiary structure of protein GrpE [25] at 100% model confidence for all four protein structures. Protein sequence alignment was undertaken with BioEdit v. 7.2.1. (Raleigh, NC, USA) [26]. The Jensen–Shannon divergence approach was employed to analyze the conservation of amino acid positions among the strains [27], and amino acid conservation was based on a relative amino acid conservation score of 75%. Single nucleotide polymorphisms (SNPs), based on phylogeny among the strains, were identified using CSI Phylogeny server v. 1.4 (Lyngby, Denmark) [28]. The WGS was aligned to the reference DSM 10599, and subsequently SNPs were called and filtered. Based on CSI phylogeny, we validated WGS reads and inferred relationships based on the concatenated alignment of SNPs.

## 3. Results

### 3.1. Wide Variations in Bacillus Species

**Figure 1:**
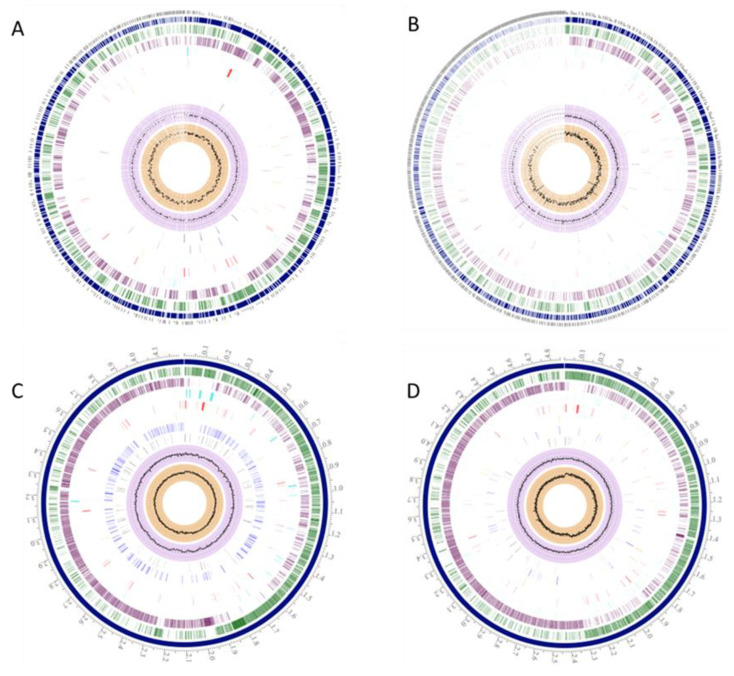
*B. sporothermodurans* SA01 (**A**), *B. oleronius* (**B**), *B. subtilis* subsp. *subtilis* str. 168 (**C**), and *B. cereus* ATCC 10987 (**D**) possess unique genomic regions in comparison with each other. The outer circle designates the genome’s coordinates in mega base pairs (Mbp). The blue circle denotes the number of contigs, and the green and purple circles represent the coding sequences (CDS), forward and reverse, respectively, with white spaces between the CDS accounting for hypothetical proteins. Non-CDS features are shown by the light blue circle. The proceeding red and orange circles represent regions encoding antimicrobial determinants and virulence factors, respectively. The two inner black circles depict the G + C contents and the skew.

To compare *B. sporothermodurans* strains (SAD, SA01, BR12, DSM 10599, B4102) with other closely related *Bacillus* species, the genomic features of *B. sporothermodurans* strains, as determined and deposited in GenBank, were compared to type strains from the *B. subtilis* group (*B. subtilis*, *B. licheniformis*, *B. amyloliquefaciens*) and the *B. cereus* group (*B. cereus*, *B. anthracis*, *B. thuringiensis*). The average genome size of the *B. sporothermodurans* strains was 3.7 Mb, on average 25% smaller than that of *B. oleronius*, 10% smaller than that of the *B. subtilis* group strains, and 30% smaller than that of the *B. cereus* group strains. The G + C content of the *B. sporothermodurans* strains was 36.08% on average, similar to that of *B. oleronius* (35%) and that of *B. cereus* group strains (35.4%), but lower than that of the *B. subtilis* group strains (45.46%). The presence of plasmids was detected only for the *B. cereus* group strains. Concerning antimicrobial resistance genes, 33 were detected in *B. sporothermodurans*, 51 in *B. subtilis*, and 46 in *B. cereus* (Figure 1).

### 3.2. Phylogenetic Analysis Depicts Distinct Clusters of B. sporothermodurans Separate from the B. subtilis Group

**Figure 2:**
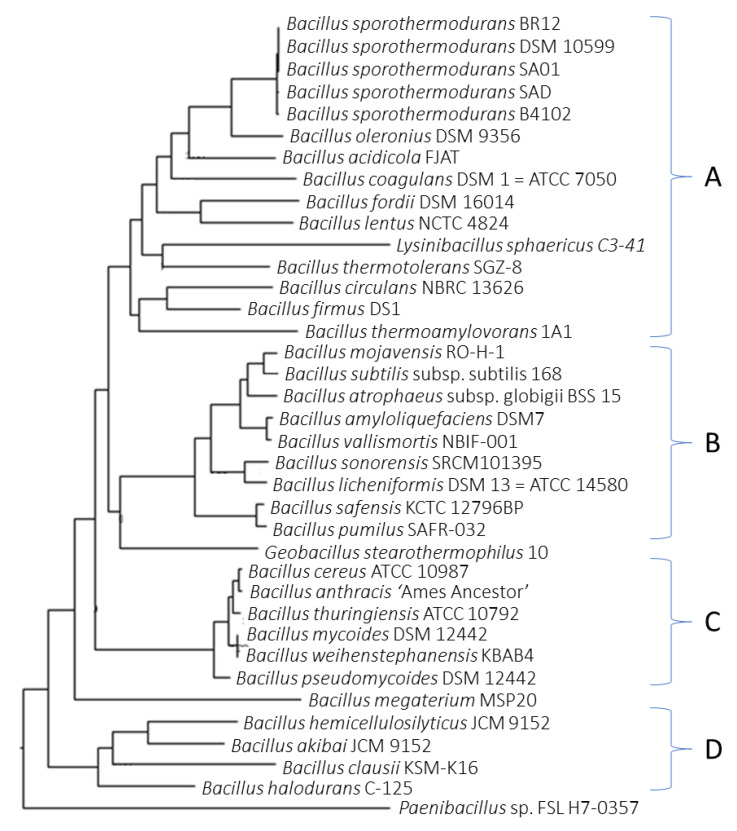
Phylogenetic analysis of *Bacillus* species showed distinct clusters for *B. sporothermodurans* and its group (**A**), *B. subtilis* group (**B**), *B. cereus* group (**C**), and selected alkaliphiles from *Bacillus* genus (**D**). The tree was constructed using the Randomized Axelerated Maximum Likelihood (RAxML) and FigTree programs.

Figure 2 shows the phylogenetic relationship of *Bacillus* species. *Bacillus oleronius* showed higher relatedness to *B*. *sporothermodurans* strains and both *B. thermotolerans* and *B. thermoamylovorans*, two highly heat-resistant bacteria grouped in the *B. sporothermodurans* cluster (cluster A). Other clusters included the *B. subtilis* group (cluster B) and the *B. cereus* group (cluster C). Cluster D mostly consisted of alkaliphiles, including *B*. *akibai* and *B*. *hemicellulosilyticus*, used in the degradation of cellulose and starch in the biotechnology industry.

### 3.3. SNP Analysis

**Figure 3:**
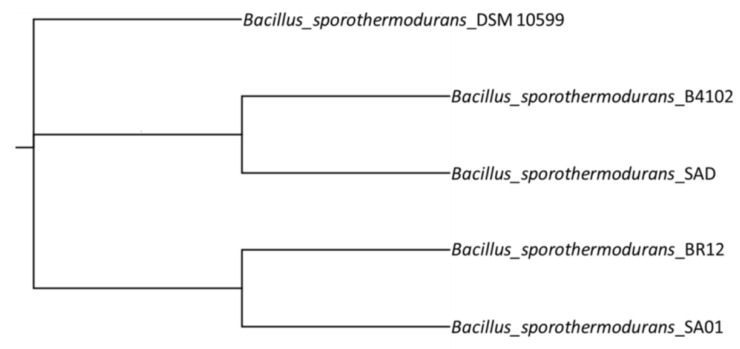
Single nucleotide polymorphism (SNP) analysis showing the phylogeny of the five *B. sporothermodurans* strains SAD, SA01, BR12, DSM 10599, and B4102, with type strain DSM 10599 as reference. The phylogenetic tree was derived from the Newick files of CSI phylogeny and processed with FigTree.

The phylogenetic tree constructed from SNP calling (Figure 3) shows the two *B. sporothermodurans* strains belonging to the HRS clones clustered together. The number of SNPs between those strains was 183, as opposed to 11,025 SNPs for the two most distant strains.

### 3.4. Protein Clusters Involved in Heat Resistance

**Figure 4:**
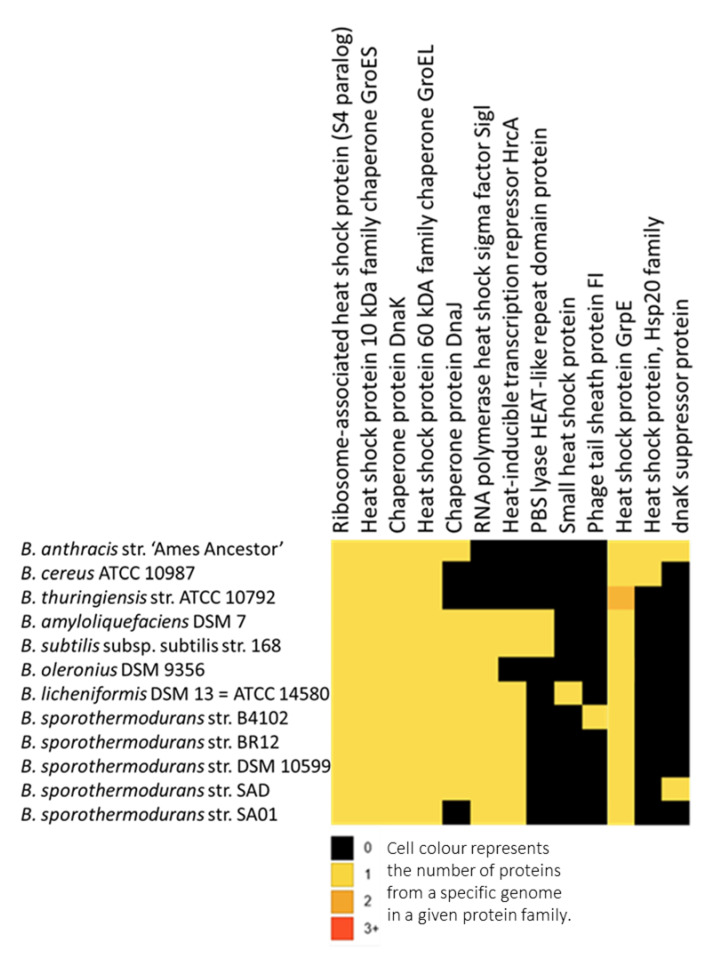
Heat map of genomes of *Bacillus* species and associated protein clusters responsible for heat resistance. The heat map was inferred using the PATRIC protein families’ utility and clustered by genomes using Pearson’s correlation and pairwise average linkage. Cell color represents the number of proteins from a specific genome in each protein family.

**Figure 5:**
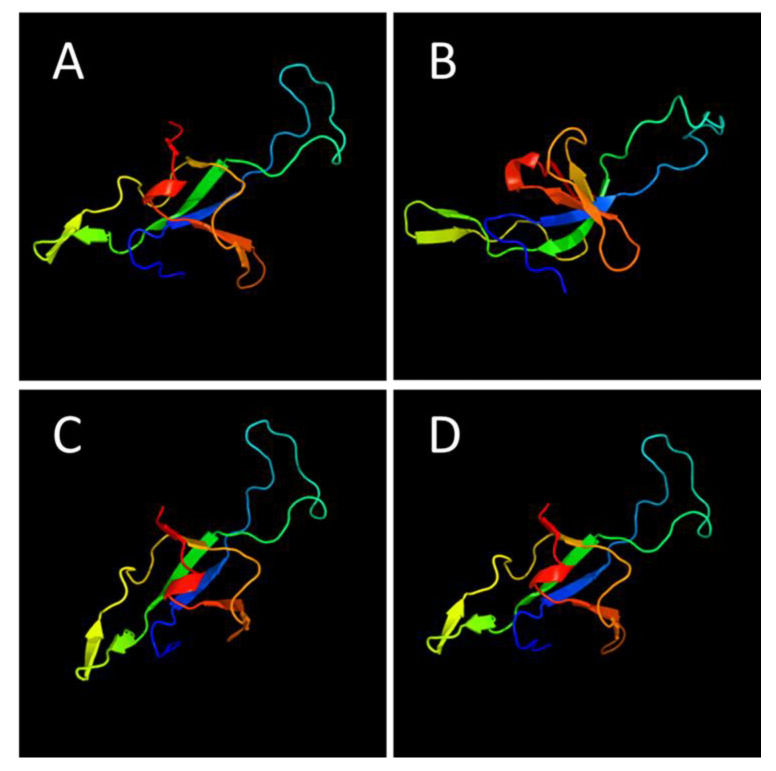
Comparison of predicted tertiary structure of the heat shock protein GrpE as identified in *B. sporothermodurans* strains SA01, SAD, BR12, DSM 10599, and B4102 (**A**), *B*. *oleronius* DSM 9356 (**B**), *B*. *cereus* ATCC 10987 (**C**), and *B*. *subtilis* subsp. *subtilis* str. 168 (**D**). Models were predicted using the Phyre2 suite of tools and at 100% model confidence for all four protein structures. Protein sequence alignment was performed with BioEdit v. 7.2.1.

The protein families identified as responsible for heat resistance are shown in Figure 4, including a total of 21,422 protein clusters, with a core of 482 protein clusters common to all 12 *Bacillus* species. *Bacillus sporothermodurans*, *B. oleronius*, *B. subtilis* group, and *B. cereus* group presented 679, 2559, 659, and 2476 clusters, respectively. *Bacillus sporothermodurans* had a total of 38, 68, and 431 clusters common to the *B. subtilis* group, *B. cereus* group, and *B. oleronius*, respectively. We highlight GrpE since it exhibited less homology between *B*. *sporothermodurans* strains and other *Bacillus*, as indicated by multiple sequence alignments. Figure 5 depicts the tertiary protein structures of GrpE from *B. sporothermodurans* and from its closest phylogenetic neighbor, *B*. *oleronius*, in comparison with *B*. *cereus* and *B*. *subtilis*. The protein structure of the four *Bacillus* species had, on average, a 64% identity with the GroES family of proteins and a 37.5% average identity with the human mitochondrial chaperonin symmetrical football complex.

**Figure 6:**
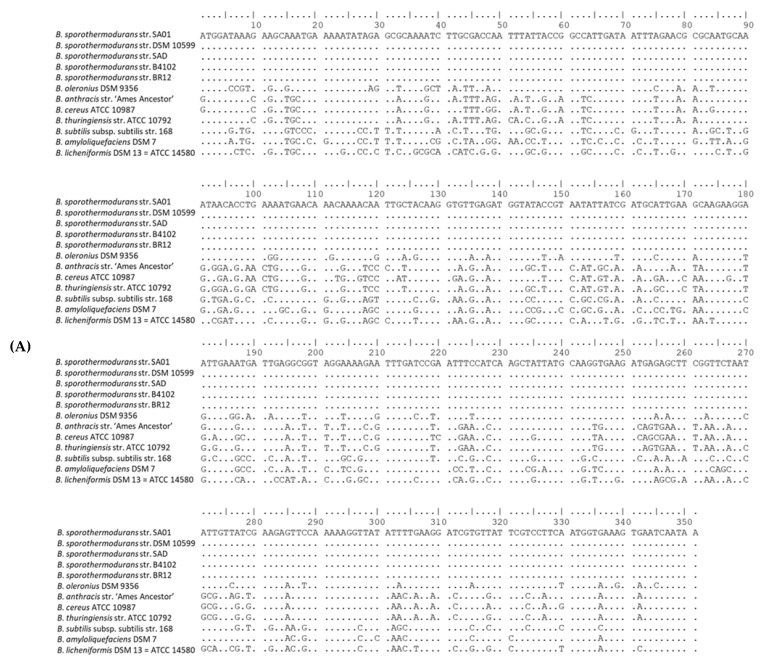

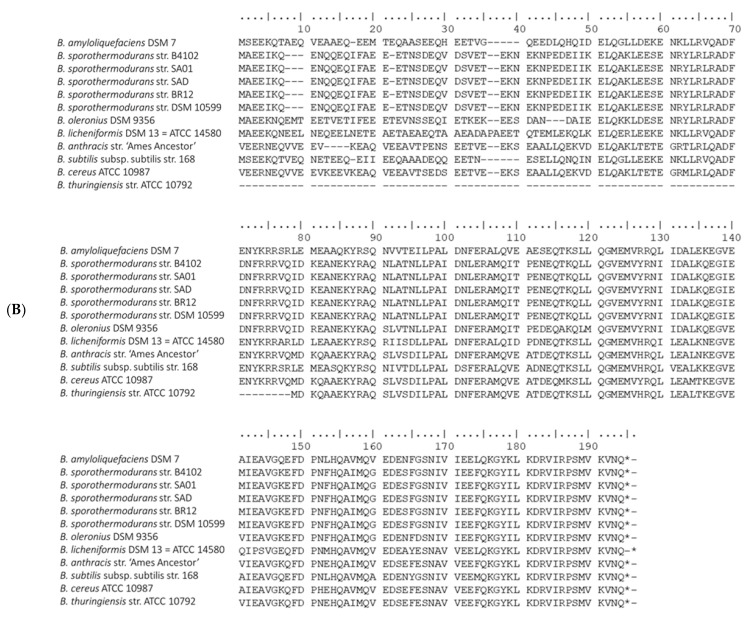
Nucleotide sequence alignment of the heat shock protein, GrpE (**A**), showing conserved regions, and the sequence alignment of its corresponding amino acid sequence (**B**), indicating the effects of the various mutations of the nucleotides on the protein sequence. Sequences were aligned with BioEdit v. 7. (**A**,**B**) were divided into four and three sections, respectively, to aid viewing.

**Figure 7:**
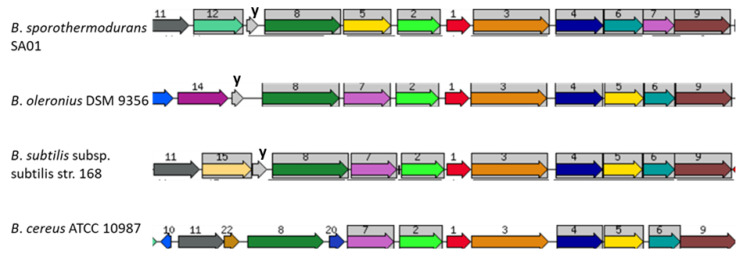
Comparison of chromosomal regions of the heat shock protein GrpE (1; red) of *B*. *sporothermodurans* SA01, *B*. *oleronius* DSM 9356, *B*. *subtilis* subsp. *subtilis* str. 168, and *B*. *cereus* ATCC 10987. Sets of genes with similar sequences are grouped with the same number and color. Genes whose relative positions are conserved in at least four other species are functionally coupled and share grey background boxes. Proteins of interest in the region are as follows: 2, heat-inducible transcription repressor HrcA; 3, chaperon protein DnaK; 4, chaperon protein DnaJ; 5, hypothetical radical SAM family enzyme in heat shock genes cluster; 6, ribosomal protein L11 methyltransferase; 7, ribosomal RNA small subunit methyltransferase; 8, translation elongation factor LepA; 9, tRNA-t(6)A37 methylthiotransferase; 10, endopeptidase spore protease Gpr; 11, SSU ribosomal protein S20p; 12, stage II sporulation protein P (SpoIIP); 14, sodium-dependent phosphate transporter; 15, transamidase GatB domain protein; 20, transcriptional regulator, HxIR family; 22, hypothetical protein; **y**, *yqe*P/*yqx*A gene.

**Figure 8:**
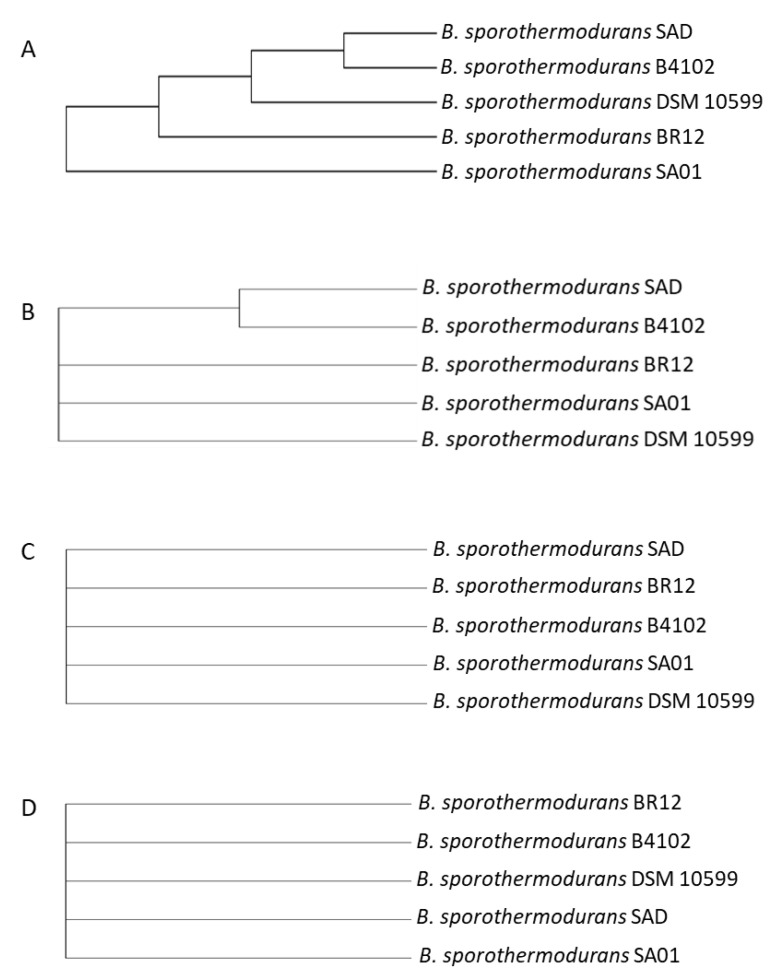
Molecular phylogenetic analysis by the maximum likelihood method of chaperon protein ClpB (**A**) and ATPases ClpC (**B**), ClpE (**C**), and ClpX (**D**). The evolutionary history was inferred by using the maximum likelihood method based on the Jones-Taylor-Thornton (JTT) matrix-based model [29]. The bootstrap consensus tree inferred from 1000 replicates was used to represent the evolutionary history of the taxa analyzed [30]. Branches corresponding to partitions reproduced in less than 50% bootstrap replicates are collapsed. Initial tree(s) for the heuristic search were obtained automatically by applying Neighbor-Join and BioNJ algorithms to a matrix of pairwise distances estimated using a JTT model, followed by selecting the topology with superior log likelihood value. The analysis involved five amino acid sequences. All positions containing gaps and missing data were eliminated. There were 862, 813, 708, and 422 positions for ClpB, ClpC, ClpE, and ClpX, respectively, in the final dataset. Evolutionary analyses were conducted in MEGA7 [22].

Figure 6 shows the nucleotide and amino acid sequence alignments of the heat shock protein GrpE, encoded by the *dna*K operon. The protein sequence alignment depicts areas of conservation and mutations in the *Bacillus* species under study, and no differences were observed among the *B. sporothermodurans* strains. Although GrpE, DnaK, and DnaJ are present in similar positions in all species, the hypothetical radical SAM family enzyme (gene 5) swaps locations with the ribosomal RNA small subunit methyltransferase (gene 7) only in *B. sporothermodurans*. Additionally, the ribosomal RNA small subunit methyltransferase in *B. sporothermodurans* consists of a 251-amino acid chain, as compared to 379 amino acids in *B*. *oleronius* and *B*. *cereus* and 367 in *B*. *subtilis*. The amino acid chain length for the hypothetical radical SAM family enzyme and ribosomal protein L11 methyltransferase in *B. sporothermodurans* was 379 and 313, respectively, as compared to 313 and 251, respectively, in *B*. *oleronius*, 312 and 257, respectively, in *B*. *subtilis*, and 313 and 250, respectively, in *B*. *cereus*. The *dna*K gene cluster is highly conserved in the *B. sporothermodurans* strains and exhibited identical amino acid length and configuration. There were no differences in the *grp*E position within the *dna*K gene cluster with respect to the *B. sporothermodurans* strains. Conserved genes were given the same number and are shown with a grey background color. The genomic arrangement of *grp*E, as well as associated genes responsible for heat shock in the *dna*K gene cluster, are shown in Figure 7. Sequence alignments and phylogenetic analysis undertaken for chaperon protein ClpB and ATPases ClpC, ClpE and ClpX showed variable regions. Regarding ClpB, there were nine variable regions out of the 862 amino acids in the *B. sporothermodurans* strains, with BR12 and SA01 sharing the same sequence, although SA01 presented two additional amino acids. The ClpC, ClpE, and ClpX exhibited one variable region each out of 813, 708, and 422 amino acids, respectively. Phylogenetic analysis using amino acid sequences for ClpB, ClpC, ClpE, and ClpX are shown in Figure 8.

## 4. Discussion

*Bacillus sporothermodurans* is an emerging highly heat-resistant spore-forming bacterial species that can significantly affect the quality of UHT and other heat-processed foods. Despite its increasing importance, limited information is available on its genetic characteristics and, most importantly, the relatedness to other *Bacillus* species of importance, especially regarding the food industry.

*Bacillus sporothermodurans* strains clustered with *B. oleronius* and other closely related *Bacillus* species, such as *B. lentus* and *B. firmus* (Figure 2, cluster A), which were initially considered as their closest relatives [31] through 16S rDNA partial sequencing. Whole genome phylogenetic analysis suggests that *B. firmus* is more distant from *B. sporothermodurans*, whereas *B. fordii* is a closer relative. *Bacillus thermoamylovorans*, another emerging highly heat-resistant *Bacillus* species isolated from milk and known to be almost as heat-resistant as *B. sporothermodurans* [32], also grouped within the *B. sporothermodurans* cluster. Similarly, the moderately heat-resistant *B. thermotolerans*, initially thought to be closely related to *B. firmus* [33], was included in cluster A, despite recent calls for it to be given a new genus, *Quasibacillus thermotolerans* [34]. The relatedness of the whole genome sequence of *B*. *thermotolerans* with *B*. *coagulans*, *B*. *firmus*, and *B*. *lentus*, as evident in the present study, supports its maintenance in the genus *Bacillus*. The categorization of bacterial populations is facilitated by using sequence similarity thresholds without being necessarily biologically relevant [35].

High heat-resistance in *B. sporothermodurans* has been attributed to the differences among strains [14,36]. Consequently, SNP evaluation, in addition to the pangenome, has been advocated for its use in tracking clonal strains. This combination of resources may help to remove the stumbling block of the high number of genes with unknown function and to provide a super-resolution view into bacterial subpopulations [7]. Given the proposed function of the pangenome and SNPs in adoptive evolution, the latter may be employed as a predictive genotypic marker to identify characteristics, such as high heat-resistance, attributed to strain differences, offering much needed insight into niche specification and adaptation [37,38]. The SNPs between the reference strain (DSM 10599) and the two strains belonging to the HRS clone, SA01 and BR12, were of 97 and 112, respectively, and 183 between BR12 and SA01. Although strain DSM 10599 is not characterized as an HRS clone feature by PCR, it is genetically closer to the two strains belonging to the HRS clone. This is because bacterial species may rely on a relatively small number of unique regions to generate variation [39]. The HRS South African SA01 strain clustered with the Brazilian BR12 strain, despite the geographic distance and apparently unconnected milk supply and production chains. Higher numbers of SNPs were found in strain B4102 (isolated from Indian curry) as opposed to strains SA01, SAD, BR12, and DSM 10599 (isolated from UHT milk), indicating a possible influence of niche adaptation on DNA sequence variation.

To maintain cellular proteostasis, cells need various chaperone pathways that enhance inherent protein folding [40]. These chaperones target misfolded, unfolded, and aggregated polypeptides for reactivation or degradation [41,42,43]. The misfolding of proteins may be a consequence of the effects of environmental stresses, such as increased heat exposure [40]. The chaperone pathways are especially important in organisms such as *B. sporothermodurans* and other *Bacillus* species exposed to increased heat conditions during food processing. The presence of disulfide bonds plays a critical role in the structural stabilization of intracellular proteins, with thermophiles normally rich in these bonds [44,45]. The response to heat shock, including such extreme conditions as thermal food processing, is mediated by heat shock proteins (HSPs). In addition to heat shock, HSPs are induced by other physiological stresses, such as exposure to cold, ultraviolet light, or during cell healing [46]. The main HSPs involved in prokaryotes are GroES, GrpE, DnaJ, GroEL, DnaK, HtpG, ClpB, ClpA, and ClpX [41,46,47,48,49]. Excluding ClpA, all the main heat shock proteins listed above are present in *B*. *sporothermodurans* and with identical amino acid sequences barring ClpB, in which the two HRS clones (SA01 and BR12) showed a high identity. The molecular chaperones above support forward protein folding, thus preventing aggregation. They are unable to facilitate protein disaggregation as aggregates form [50]. However, ClpB has the remarkable ability to rescue stress-damaged aggregated proteins [50]. This is achieved by the extraction of polypeptides from aggregates through forced unfolding and translocation via the ClpB central cavity and by the subsequent release for chaperone-mediated refolding [51]. The difference in the sequence of ClpB from the two HRS strains deserves further analyses to evaluate their possible influences on the highly heat-resistant phenotype. In fact, HSP100/Clp proteins are ATP-dependent chaperones that transfer proteins into the chamber of an associated barrel-like protease complex (ClpP, ClpQ) [52]. These ATPases, including ClpA, ClpC, ClpE, ClpX, and ClpY, deliver partially unfolded substrates to ClpP and ClpQ, the corresponding proteolytic component [52,53]. This process is crucial to highly heat-resistant *Bacillus* spp. during periods of stress and sporulation [54]. Previous studies identified four Clp ATPases (ClpC, ClpE, ClpX, and ClpY) [52,54] in the *B*. *subtilis* group. In this study, the coding sequence for ClpA was identified in addition to the previous Clp ATPases for *B*. *subtilis*, but not for *B*. *sporothermodurans* strains. The *B*. *cereus* group used had an identical complement of ATPases in relation to the *B*. *subtilis* group. *Bacillus sporothermodurans* and *B*. *oleronius* exhibited coding sequences for ClpC, ClpE, and ClpX. Furthermore, ClpB was identified in *B*. *subtilis*, *B*. *sporothermodurans*, and *B*. *oleronius*. The ClpB is not involved with proteases and rather works with the chaperones DnaJ, DnaK, and GrpE to repair heat-induced protein damage [55]. The ClpX targets specific proteins for degradation directly or with substrate-specific adaptor proteins. Thus, the ClpB, ClpA, ClpX protein complex facilitates the tolerance to extreme temperatures in various prokaryotes by remodeling and degrading aggregated proteins [40]. Specifically, the ClpX and ClpP protein group plays a role in cell viability at heat stress under conditions of limiting levels of the DnaK system [53].

Apart from HSP20 (present only in the *B. cereus* group), heat-inducible transcription repressor HrcA (detected in *B. subtilis* and *B. sporothermodurans*) and DnaK suppressor protein (detected in *B. amyloliquefaciens* and *B. subtilis*), the remainder of the genes of the *dna*K cluster, had homologs in all *Bacillus* groups evaluated. The *hrc*A is the first cistron of the *B. subtilis dna*K operon and encodes a negative regulator of class I heat shock genes (*dna*K and *gro*E operons) [46,56]. The DnaK, DnaJ, and GrpE form a cellular chaperone machinery involved in the repair of heat-induced protein damage [48]. Many of these proteins shared amongst the *Bacillus* species are homologs of each other, with differences along the amino acid chain. In this sense, Figure 6 depicts nucleotide and amino acid alignment of GrpE, a chaperone part of the *dna*K operon that, in conjunction with GroEL (a 60-kDa family chaperone), is responsible for the renaturation of heat-denatured proteins [48]. Differences in the arrangement of genes in the chromosomal region of *grp*E among the *Bacillus* species studied (Figure 7) are evident, but, in fact, the effects on the heat resistance or other biological functions of the change between the hypothetical radical SAM family enzyme and the ribosomal RNA small subunit methyltransferase in *B. sporothermodurans* are not known. The hypothetical radical SAM family enzyme may modulate the action of *hrc*A in *B. sporothermodurans*. Indeed, it has been shown that HrcA acts as a thermo-sensor in *B*. *subtilis* and *B*. *thermoglucosidasius*, the latter possessing significantly higher heat resistance than the former [57]. Another difference is the presence of a stage II sporulation protein P gene (SpoIIP; region 12 of Figure 7) only in *B*. *sporothermodurans*, placed just upstream of *yqe*P. The SpoIIP may be involved in heat resistance enhancement, as it has been identified as an autolysin with peptidoglycan hydrolase activity; it also participates in the engulfment process during sporulation [58]. These differences may well involve transcription regulation and protein protection mechanisms, resulting in differences in resistance to stresses, such as heat shock.

Besides the sequence variation in the chromosomal region of *grp*E, the predicted tertiary protein structure of GrpE from the *Bacillus* species studied showed differences. The GrpE structure within *B*. *sporothermodurans* strains (HRS and non-HRS) showed no differences and, as such, cannot be implied in the difference in high heat resistance between these strains. In addition, GrpE from *B. sporothermodurans* and *B*. *oleronius* presented almost identical amino acid chains, only differing on the 16th amino acid, where the former’s alanine is replaced by threonine in the latter. The detection of ‘pockets’, often found to harbor active sites along the protein structure, identified 13 likely amino acid active sites in the GrpE protein for both *B. sporothermodurans* and *B*. *oleronius*. On the other hand, *B*. *cereus* and *B*. *subtilis* exhibited 38 and 12 pockets, respectively. The high numbers of likely amino acid active sites in *B*. *cereus* may be correlated with roles in enzymatic reactions involving its numerous virulence genes, since GrpE is associated with virulence [47].

## 5. Conclusions

Comparative analysis of *B. sporothermodurans* with other *Bacillus* species showed the close relatedness of these *Bacillus* species. Proteins implicated in heat resistance showed high levels of similarity in barring GrpE, which has unique substitutions that could infer different phenotypic responses to *B*. *sporothermodurans* with respect to heat resistance. These proteins, however, do not offer compelling differences between the HRS and non-HRS strains of *B*. *sporothermodurans*, which could unambiguously explain their specific characteristics regarding thermal resilience, although the possible involvement of the SpoIIP and ClpB can be highlighted. Further laboratory studies involving mutant and wild type strains of *B*. *sporothermodurans* are required to confirm the phenotypic effects of these genetic variations.
